# Study of Single Crystal and X-Ray Detector Performance of Ti^3+^: *β*-Ga_2_O_3_

**DOI:** 10.3390/ma19112417

**Published:** 2026-06-05

**Authors:** Boyang Chen, Xinyu Liu, Yiyuan Liu, Zeliang Gao, Zhitai Jia, Wenxiang Mu

**Affiliations:** 1 State Key Laboratory of Crystal Materials, Institute of Novel Semiconductors, Institute of Crystal Materials, Shandong University, Jinan 250100, China; 202320601@mail.sdu.edu.cn (B.C.); 202314073@mail.sdu.edu.cn (X.L.); 202114039@mail.sdu.edu.cn (Y.L.); z.jia@sdu.edu.cn (Z.J.); 2 Shenzhen Research Institute of Shandong University, Virtual University Park in South District, Shenzhen 518057, China

**Keywords:** semiconductor, Ga_2_O_3_, EFG method, X-ray detector

## Abstract

**Highlights:**

- For the first time, a high-quality Ti^3+^-doped *β*-Ga_2_O_3_ single crystal was successfully grown via the EFG method.
- The photoelectric properties of *β*-Ga_2_O_3_ were effectively modulated via Ti^3+^ doping, achieving a moderate semi-insulating state and a reduced bandgap.
- Ti^3+^-doped *β*-Ga_2_O_3_ crystal was fabricated into X-ray detectors with a metal–semiconductor–metal (MSM) structure, demonstrating superior detection performance.

**Abstract:**

Gallium oxide (Ga_2_O_3_) is emerging as a promising material for X-ray detectors due to its high sensitivity, high melting point, and stable physicochemical properties. However, intrinsic background shallow donors in raw materials hinder the preparation of high-resistance intrinsic crystals, making doping essential to tailor electrical properties. This study grew Ti^3+^-doped *β*-Ga_2_O_3_ single crystals via the Edge-defined Film-fed Growth (EFG) method using Ti_2_O_3_ as a dopant, achieving high resistivity and a moderate reduction in bandgap. High-resolution X-ray diffraction (HRXRD) showed a rocking curve full width at half maximum (FWHM) of 96.50 arcsec. Compared with the unintentionally doped (UID) crystal, the bandgap exhibited a slight reduction, decreasing from 4.76 eV to 4.59 eV. In the infrared transmission spectra, the onset wavelength of the decrease in transmittance for the Ti^3+^: *β*-Ga_2_O_3_ crystal showed a distinct redshift relative to that of the UID crystal, indicating effective suppression of free electrons within the crystal. X-ray photoelectron spectroscopy (XPS) revealed that Ti^3+^ incorporation minimally affected the valence states of Ga and O or the Ga/O ratio, with no significant shift in valence band maximum (EVBM). A metal–semiconductor–metal (MSM) structured X-ray detector fabricated on polished Ti^3+^: *β*-Ga_2_O_3_ (100) substrate with Ti/Au electrodes exhibited a peak sensitivity of 943.16 μC/(Gy·cm^2^) at 40 V bias and 2.944 μGy/s dose rate, surpassing the upper sensitivity limit reported for semi-insulating doping bulk *β*-Ga_2_O_3_ detectors. The rise and fall times were 0.23 s and 0.30 s, respectively, with a minimum detectable limit (MDL) of 164.26 nGy/s, demonstrating its potential for high-performance X-ray detection applications.

## 1. Introduction

X-ray as a versatile form of high-energy electromagnetic waves, play an indispensable role across various fields including medical imaging, non-destructive testing, and material analysis. In everyday contexts, X-rays are most commonly encountered during medical examinations using X-ray radiography and computed tomography (CT) scans [1]. However, due to their high energy, excessive exposure to X-ray radiation, whether in terms of intensity or cumulative dose, can pose significant health risks. To mitigate potential harm, it is crucial to minimize the radiation dose during diagnostic procedures, which, in turn, demands detectors with enhanced sensitivity to accurately capture low-dose X-ray signals. Semiconductor-based X-ray detectors, such as those made from amorphous selenium (α-Se) or cadmium zinc telluride (CZT), have been successfully commercialized for medical applications due to their ability to generate sufficiently strong signals under low radiation doses [2,3]. Nevertheless, these materials face inherent limitations that constrain further development. For example, α-Se exhibits relatively low sensitivity compared to other semiconductor materials and is prone to crystallization at elevated temperatures, thereby limiting its operational stability. Meanwhile, CZT crystals present challenges in terms of growth complexity, which affects production yield and leads to higher costs. To address these issues and meet the growing demand for improved performance, the exploration of new detector materials is essential. *β*-Ga_2_O_3_, a wide-bandgap semiconductor, has emerged as a promising candidate for X-ray detection. Notably, *β*-Ga_2_O_3_ possesses one of the highest X-ray absorption capacities among wide-bandgap semiconductors, a mere 1.5 mm thickness is sufficient to absorb 90% of 50 keV X-rays [4]. With a bandgap of 4.8 eV, a melting point of 1793 °C, and excellent physicochemical stability, *β*-Ga_2_O_3_ demonstrates remarkable tolerance to high voltages and temperatures, making it well-suited for harsh radiation environments. The electrical properties of *β*-Ga_2_O_3_ can be effectively tuned through defect engineering: introducing shallow donors such as Si or Sn increases free electron concentration [5,6], thereby reducing resistivity and on-state resistance in devices, while deep-level acceptors like Fe or Mg introduce trapping centers that compensate free carriers, increase resistivity, and suppress dark current [7,8]. This capability to tailor electrical characteristics enables the fabrication of *β*-Ga_2_O_3_-based detectors with high sensitivity, improved signal-to-noise ratio, and lower detection limits, positioning *β*-Ga_2_O_3_ as a highly promising material for next-generation X-ray detection applications.

For unintentionally doped (UID) *β*-Ga_2_O_3_, the presence of a certain concentration of free carriers makes it challenging to achieve high-performance X-ray detectors with satisfactory breakdown strength and electrical properties. These free carriers primarily originate from residual shallow-level donors such as Si and Sn impurities introduced during crystal growth. As previously discussed, these elements form defect levels close to the conduction band of *β*-Ga_2_O_3_, allowing electrons to be easily excited into the conduction band under an applied bias, thereby generating unwanted leakage current that interferes with the detection signal. To mitigate this issue, semi-insulating doping can be employed to increase the material’s resistivity. X-ray detectors fabricated from semi-insulating doped *β*-Ga_2_O_3_ crystals have demonstrated improved performance and enhanced breakdown strength [9,10]. In particular, Ni-doped *β*-Ga_2_O_3_ has yielded the highest reported detection sensitivity of 633.63 μC/Gy·cm^2^ among semi-insulating bulk *β*-Ga_2_O_3_ single crystals [11]. However, while deep-level acceptors can effectively suppress dark current by trapping free carriers, such trapping is non-selective. These deep-level centers also capture photogenerated carriers produced under X-ray irradiation, thereby reducing photocurrent and limiting the ultimate sensitivity of the device.

To overcome this limitation, bandgap engineering offers an alternative approach. Given that the ionization energies of shallow donors like Si and Sn are as low as 15–30 meV and 7.4–60 meV, respectively [12], modifying the conduction band position can effectively suppress carrier excitation. Alloying Ga_2_O_3_ with other metal oxides enables tunable bandgaps. For instance, Krueger et al. reported that (Al_x_Ga_1−x_)_2_O_3_ solid solutions can exhibit bandgaps ranging from 4.8 eV to 8.7 eV, from that of *β*-Ga_2_O_3_ to that of α-Al_2_O_3_, while maintaining the β-phase for x < 0.7 [13]. Based on the phenomenon that the incorporation of Al^3+^ can widen the bandgap of Ga_2_O_3_, Li et al. incorporated Al^3+^ into *β*-Ga_2_O_3_ to upshift the conduction band minimum, effectively inhibiting electron excitation and reducing dark current. At 15% Al doping, the detector achieved a sensitivity of 851.6 μC/Gy·cm^2^, the highest reported among bulk Ga_2_O_3_ crystal-based X-ray detectors [14]. Nevertheless, high Al incorporation can induce lattice distortion and degrade crystal quality. Thus, although both doping and bandgap engineering improve detector performance, each has limitations, necessitating further exploration of optimized doping strategies in Ga_2_O_3_.

The operation of a Ga_2_O_3_-based MSM X-ray detector involves the absorption of X-rays by the substrate, generating electron–hole pairs, which are then separated by an external electric field to produce a measurable photocurrent [15]. Based on this rationale, the X-ray detection capability of *β*-Ga_2_O_3_ can be enhanced by synergistically combining electrical property regulation with bandgap engineering. This integrated approach entails introducing deep-level defects to suppress the free carriers contributed by background shallow donors, while concurrently reducing the bandgap to generate more photogenerated carriers. Furthermore, owing to the high resistivity of the crystal, the reduction in bandgap does not lead to a significant increase in dark current. This strategy ultimately results in improved overall detection performance [16]. Alloying *β*-Ga_2_O_3_ with wider-bandgap Al_2_O_3_ increases the bandgap, whereas combining it with smaller-bandgap oxides can reduce it. For example, Lin et al. synthesized (Ga_1−x_In_x_)_2_O_3_ thin films via sputtering, tuning the bandgap from 4.94 eV to 3.42 eV [17]. Similarly, Li et al. grew *β*-Ga_2_O_3_ crystals with 9% and 15% In using the optical floating zone method, observing bandgap reduction, though at the cost of increased carrier concentration [18]. Incorporating Ti can also reduce the bandgap; Wang et al. studied Ti^3+^: *β*-Ga_2_O_3_ crystals grown by the optical floating zone method using Ti_2_O_3_ as a dopant [19]. Although the bandgap of Ti_2_O_3_ remains debated, the reported values were all lower than *β*-Ga_2_O_3_ [20,21], its incorporation clearly reduces the bandgap of Ga_2_O_3_. Additionally, Ti doping introduces multiple deep-level defects, which have a high activation energy and can pin the Fermi level near the mid-gap, rendering the crystal semi-insulating, a desirable trait for detector materials. Therefore, Ti^3+^: *β*-Ga_2_O_3_ represents a promising candidate for further exploration in high-sensitivity X-ray detection.

In this work, a Ti^3+^: *β*-Ga_2_O_3_ single crystal using Ti_2_O_3_ as the dopant was grown by the EFG method. After evaluating the crystal quality by high-resolution X-ray diffraction (HRXRD), the crystal was subjected to double-sided chemical mechanical polishing. The bandgap and electrical properties were analyzed using ultraviolet–visible–near-infrared transmission spectroscopy and compared with those of an undoped (UID) crystal. The results indicated a reduction in the bandgap of Ti^3+^: *β*-Ga_2_O_3_ and the emergence of semi-insulating behavior. XPS was further employed to analyze the elemental distribution, valence states, and EVBM, clarifying the specific effects of Ti^3+^ incorporation and confirming that the decrease in conduction band position is the primary reason for the bandgap narrowing. Subsequently, Ti/Au electrodes were deposited on both sides of the substrate to fabricate an MSM-structured X-ray detector. The detector performance was systematically investigated, including its photocurrent, sensitivity, signal-to-noise ratio, and detection limit. Ultimately, a breakthrough in sensitivity was achieved, reaching 943.16 μC/Gy·cm^2^, setting a new record for bulk single-crystal detectors.

## 2. Materials and Methods

*β*-Ga_2_O_3_ bulk crystals were grown by the EFG method. 5N Ga_2_O_3_ powders from Aluminum Corporation of China (Beijing, China) and 4N Ti_2_O_3_ from Shanghai Aladdin Biochemical Technology Co., Ltd. (Shanghai, China). were mixed for over 60 h to serve as the raw materials. The crystal was grown in a JMD-800 furnace produced by Jinan Jinmaden Automation Technology Co., Ltd. (Jinan, China), with the growth atmosphere consisting of 50% Ar and 50% CO_2_ at a pressure slightly above atmospheric. The growth process proceeded through four stages: seeding, shouldering, isodiametric growth, and termination. An iridium crucible measuring Φ80 (R) × 60 (H) mm^3^ and a die with dimensions of 50 (L) × 4 (W) mm^2^ were used. During growth, the pulling speed was maintained between 5 and 10 mm/h. After growth, the temperature was programmed to decrease slowly to room temperature at a rate of 20–30 °C/h. The as-grown crystal wafer was subjected to double-side chemical mechanical polishing using a POLI-500 system from Beijing TSD Semiconductor Equipment Co., Ltd. (Beijing, China). The crystalline quality of the Ti^3+^: *β*-Ga_2_O_3_ single crystal was characterized by high-resolution X-ray diffraction (HRXRD) using a Bruker-AXS D5005HR diffractometer from Bruker AXS SE (Bruker, Karlsruhe, Germany) with Cu-Kα radiation. The transmittance spectra along the [100] direction at room temperature were recorded on a Hitachi UV–Vis–NIR spectrometer manufactured by Hitachi High-Tech Corporation (Tokyo, Japan), witch covering wavelengths from 200 to 12,000 nm. XPS measurements, including the E_VBM_, were performed on the Ti^3+^: *β*-Ga_2_O_3_ (100) plane using a PHI 5000 Versaprobe II system by ULVAC-PHI, Inc. (Chigasaki, Japan). The X-ray detection performance was evaluated in a dark lead-shielded box at room temperature. A tungsten anode X-ray source was employed, with the irradiation dose rate adjustable by varying the tube voltage and current.

## 3. Results and Discussion

**Figure 1:**
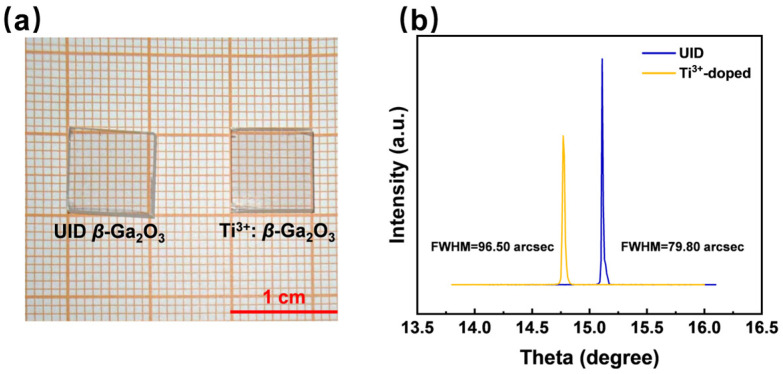
(**a**) Photograph of the UID and Ti^3+^: *β*-Ga_2_O_3_ single crystal grown by the EFG method. (**b**) Rocking curves of Ti-doped and UID *β*-Ga_2_O_3_ single crystals.

Uniformly mixed Ti_2_O_3_ and Ga_2_O_3_ powders were used to grow a Ti^3+^: *β*-Ga_2_O_3_ crystal via the EFG method. The (100)-oriented wafer of UID and Ti^3+^: *β*-Ga_2_O_3_ processed and chemically and mechanically polished were shown in Figure 1a. Although an increased concentration of deep-level acceptors can effectively suppress the dark current of the detector during operation and enhance its breakdown voltage, excessive doping may induce severe lattice distortion, degrading crystal quality. Moreover, a higher density of deep-level traps can lead to strong space-charge effects under high electric fields [22], adversely affecting key detector performance parameters such as sensitivity and response time. Therefore, a doping concentration of 1 × 10^18^ cm^−3^ was selected for this growth. This concentration was chosen to compensate for the free electrons provided by the inherent shallow donors within the crystal, while minimizing the impact on crystal quality and avoiding the introduction of an excessive number of deep-level defects. The quality of the exfoliated surface was rapidly characterized by HRXRD and compared with the (100) plane of a UID crystal. The results were presented in Figure 1b. For the UID crystal, the (400) diffraction peak showed a rocking curve FWHM of 79.80 arcsecs and a peak position at 15.11 degrees. In contrast, the Ti^3+^: *β*-Ga_2_O_3_ crystal exhibited an FWHM of 96.50 arcsecs and a peak position at 14.77 degrees for the (400) reflection. The decrease in the θ angle corresponding to the (400) diffraction peak in the Ti-doped sample, compared to the UID sample, is attributed to lattice distortion caused by Ti^3+^ substitution for Ga^3+^. Since the ionic radius of Ti^3+^ is larger than that of Ga^3+^, the predominant effect is lattice expansion, leading to an increase in the interplanar spacing. According to Bragg’s law, when the incident X-ray wavelength is fixed, the interplanar spacing is inversely proportional to the diffraction angle θ. Therefore, the (100) diffraction peak of Ti^3+^: *β*-Ga_2_O_3_ shifts to a lower angle compared to that of the UID crystal.

**Figure 2:**
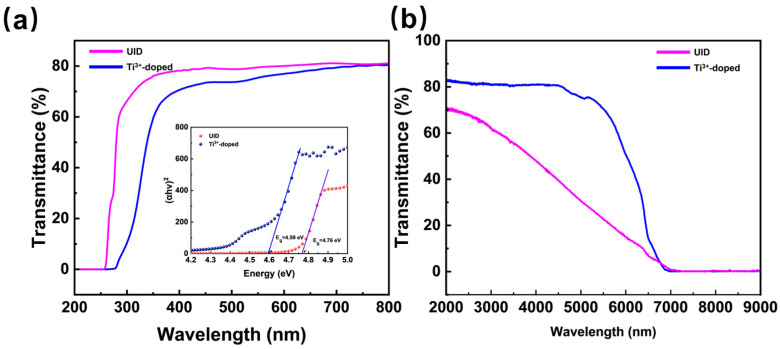
(**a**) UV–vis transmittance spectra and bandgap, and (**b**) infrared transmittance spectra of Ti-doped and UID *β*-Ga_2_O_3_.

UV–vis transmittance spectroscopy provides an efficient, non-destructive, and rapid method for measuring the optical properties and bandgap of UID and Ti^3+^: *β*-Ga_2_O_3_ crystals. The corresponding results are shown in Figure 2a. The UV absorption trend of the Ti^3+^: *β*-Ga_2_O_3_ crystal was consistent with that of the UID *β*-Ga_2_O_3_. When the wavelength was shorter than the UV absorption edge, the transmittance of UV light was relatively low. As the wavelength increased, the transmittance gradually rose, reaching a maximum of approximately 80% without subsequent decay. This behavior distinctly differed from that typically observed in conductivity-doped crystals. However, a clear shift in the UV absorption edge was observed between the two samples. The absorption edge for the (100) plane of the UID crystal was measured at 261.6 nm, which was lower (i.e., at a shorter wavelength) than the 270.1 nm observed for the Ti^3+^: *β*-Ga_2_O_3_ crystal. The position of the UV absorption edge is directly related to the bandgap. A narrower bandgap corresponds to a longer absorption edge wavelength (i.e., a redshift). Equation 1 describes the relationship between the absorption coefficient and the bandgap for Ga_2_O_3_ [23,24]:

αhν = A(hν − E_g_)^1/2^ (1)

where α is the absorption coefficient, hν represents the photon energy, A is a proportionality constant, and Eg denotes the optical bandgap. After conversion using the Tauc plot method, the optical bandgaps of the UID and Ti^3+^: *β*-Ga_2_O_3_ crystals were linearly derived to be 4.76 eV and 4.59 eV, respectively, as shown in Figure 2a. Compared with the UID crystal and common semi-insulating β-Ga_2_O_3_ single crystals doped with elements such as Mg or Fe, the bandgap of the Ti-doped crystal was significantly narrowed. Table 1 summarizes the bandgaps for various common dopants and different crystal planes. In general, the (010) plane typically exhibits the narrowest bandgap in *β*-Ga_2_O_3_, while the (100) and (001) planes possess considerably wider bandgaps. The bandgap of the Ti^3+^: *β*-Ga_2_O_3_ (100) plane was reduced to a level comparable to that of the (010) plane, indicating that Ti incorporation effectively modulated the energy band structure.

*β*-Ga_2_O_3_ can efficiently absorb X-rays. Due to the high energy of X-rays, electron-hole pairs are generated inside the gallium oxide. By applying a suitable bias voltage, a detectable electrical signal can be obtained, thereby enabling X-ray detection. For X-ray detectors made from such wide-bandgap semiconductors, the magnitude of the bandgap directly influences the generation efficiency of photogenerated carriers. Equation (2) gives the expression for the relationship between the minimum energy required to produce an electron-hole pair in a semiconductor material, i.e., the minimum ionization energy ΔE, and the bandgap E_g_ [26,27]:

ΔE = 2.8E_g_ + 0.6 (2)

According to the formula, the ionization energy is positively correlated with the bandgap. Therefore, a reduction in the bandgap leads to a corresponding decrease in the required ionization energy. In other words, materials with a narrower bandgap require lower energy to generate photocarriers. Thus, under identical irradiation dose and intensity, a material with a narrower bandgap can generate more photocarriers. However, reducing the bandgap was found to increase the dark current. Since the extent of bandgap narrowing was limited, merely considering bandgap engineering could not provide a significant enhancement to the detection capability of *β*-Ga_2_O_3_; instead, it might even degrade the detection performance due to the elevated dark current. Nevertheless, the incorporation of Ti^3+^ simultaneously introduced a certain concentration of deep-level defects, which transformed the electrical properties of the crystal into a semi-insulating state. Figure 2b indicated the infrared (IR) transmittance spectra of the UID and Ti^3+^: *β*-Ga_2_O_3_ crystals. Since free electrons in *β*-Ga_2_O_3_ crystals exhibit significant absorption in the IR region, the IR transmittance spectrum serves as an effective indicator of the free electron concentration within the crystal. Specifically, a high electron concentration leads to stronger absorption of IR light, resulting in a pronounced attenuation of the IR transmittance spectrum. This is often accompanied by a blue shift of the infrared absorption edge, meaning the corresponding cutoff wavelength decreases significantly. Conversely, when the free electron concentration is relatively low, the material’s IR absorption weakens, causing the onset wavelength of transmittance attenuation to shift to a longer wavelength. The transmittance of the UID crystal began to decrease at a wavelength of approximately 2100 nm, whereas the Ti-doped sample showed no significant decrease until around 4500 nm. This indicates that Ti_2_O_3_ doping effectively introduces deep-level defects into the crystal. These defects trap background free electrons, thereby effectively reducing the free carrier concentration and transforming the electrical properties of the crystal to a semi-insulating state. Compared with Ni^2+^: *β*-Ga_2_O_3_ crystal, the Ti^3+^: *β*-Ga_2_O_3_ crystal exhibited a shorter-wavelength infrared absorption cutoff edge, indicating a higher electron concentration and consequently a lower resistivity than those of Ni^2+^: *β*-Ga_2_O_3_ crystal [11]. In summary, characterization via UV–Vis–IR transmittance spectroscopy confirms that the optoelectronic properties of the Ti^3+^: *β*-Ga_2_O_3_ crystal meet the design requirements for the intended device application.

Although a significant reduction in the bandgap of our as-grown Ti^3+^: *β*-Ga_2_O_3_ was observed, the extent of the decrease was somewhat smaller compared to previous reports, which may be related to the relatively low Ti concentration introduced during growth and the concentration of oxygen vacancies (V_O_). In bandgap engineering studies of *β*-Ga_2_O_3_, the degree of bandgap modulation is directly correlated with the concentration of the introduced impurity ions. Compared with the work by Wang et al., our doping concentration was 1 × 10^18^ cm^−3^ (0.00185 wt%), which is substantially lower than their 0.2 wt%. Consequently, its impact on the bandgap is less pronounced. Furthermore, the incorporation of Ti^3+^ has been confirmed to induce lattice expansion, which, in itself, can alter the material’s bandgap, and the degree of lattice expansion varies with different doping concentrations. On the other hand, according to research by Chen et al., increasing the concentration of V_O_ in the crystal can intentionally modify the lattice disorder and lower the energy of the E_VBM_, meaning the valence band moves closer to the Fermi level, thereby reducing the bandgap [28]. To analyze the extent to which the V_O_ concentration, influenced by Ti^3+^ incorporation, affects the bandgap of Ti^3+^: *β*-Ga_2_O_3_, we performed X-ray XPS on both UID and Ti^3+^: *β*-Ga_2_O_3_. Analysis of the Ga 3d and O 1s XPS peaks provides specific information about the surface elemental composition and chemical states of the crystals.

**Figure 3:**
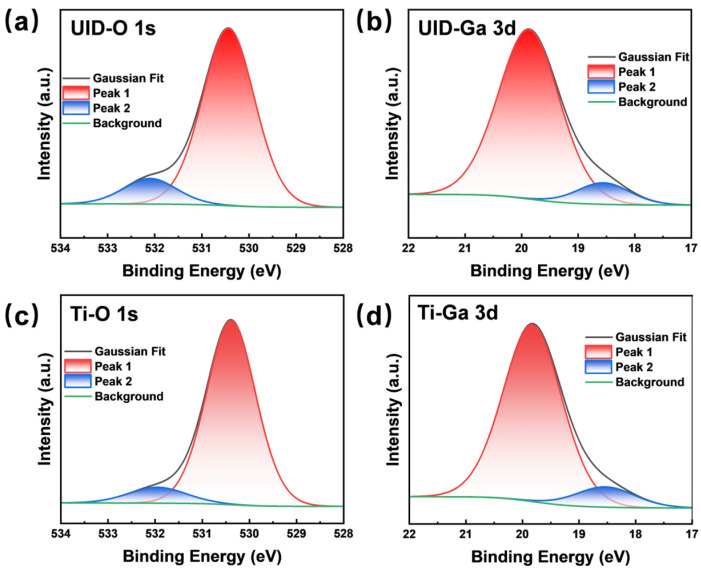
High-resolution spectra of (**a**) O 1s and (**b**) Ga 3d for Ti^3+^: *β*-Ga_2_O_3_ and (**c**) O 1s and (**d**) Ga 3d for Ti^3+^: *β*-Ga_2_O_3_.

The XPS results for both samples are shown in Figure 3, with all peak positions calibrated against the C 1s peak at 284.8 eV. Figure 3a displays the Ga 3d spectrum of the UID crystal. Peak deconvolution revealed two Gaussian peaks at different binding energies. The more intense peak at 19.92 eV corresponds to Ga^3+^, which is the dominant state in gallium oxide. The peak at 18.64 eV confirms the presence of a certain amount of Ga^+^ within the crystal [29]. Figure 3b shows the Ga 3d spectrum of the Ti^3+^: *β*-Ga_2_O_3_ crystal. Similarly, deconvolution yielded two Gaussian peaks. Compared to the UID crystal, the peak positions shifted to lower binding energies at 19.82 eV and 18.53 eV, respectively. This indicates that Ti incorporation altered the crystal’s microstructure to some extent, likely causing a change in gallium vacancy (V_Ga_) concentration, which led to the peak shift. However, based on the relative positions, Ga still exists primarily as Ga^3+^ and minor Ga^+^, confirming that Ti doping did not alter the valence state of Ga. Figure 3c and Figure 3d present the high-resolution O 1s spectra for the UID and Ti^3+^: *β*-Ga_2_O_3_ crystals, respectively. The asymmetric line shapes indicate that, in addition to lattice oxygen, a certain concentration of V_O_ exists within the crystals. Deconvolution of the O 1s spectra also produced two Gaussian peaks. For the UID crystal, the two peaks were located at 530.43 eV and 532.10 eV. The smaller peak at 532.10 eV is attributed to V_O_. For the Ti-doped crystal, the deconvoluted O 1s peaks were located at 530.39 eV and 531.95 eV. The O 1s spectra of the UID and Ti-doped samples were very similar, with no additional peaks appearing. However, the relative intensities of the corresponding peaks differed, suggesting that Ti incorporation influenced the V_O_ concentration. By integrating the two deconvoluted Gaussian peaks in the O 1s spectra and comparing their areas, a simple semi-quantitative assessment of the oxygen vacancy content can be made. The calculated V_O_ concentration was 12.75% for the UID crystal and decreased to 9.16% for the Ti^3+^-doped crystal. If bandgap reduction were achieved by increasing V_O_ concentration, this value would be expected to rise. Therefore, the decrease in the bandgap of Ti^3+^: *β*-Ga_2_O_3_ is not related to the change in V_O_ concentration. Moreover, the reduction in V_O_ concentration can effectively minimize its adverse impact on the response time of subsequently fabricated detectors. Quantitative calculation of the Ga/O ratio was performed by integrating the peak areas of the Ga 3d and O 1s XPS peaks and applying their respective relative sensitivity factors (RSFs), which are 0.43 for Ga 3d and 0.733 for O 1s [30]. The calculated Ga/O ratios were 0.633 for the UID crystal and 0.624 for the Ti^3+^: *β*-Ga_2_O_3_ crystal under the same growth conditions, showing little difference [31].

**Figure 4:**
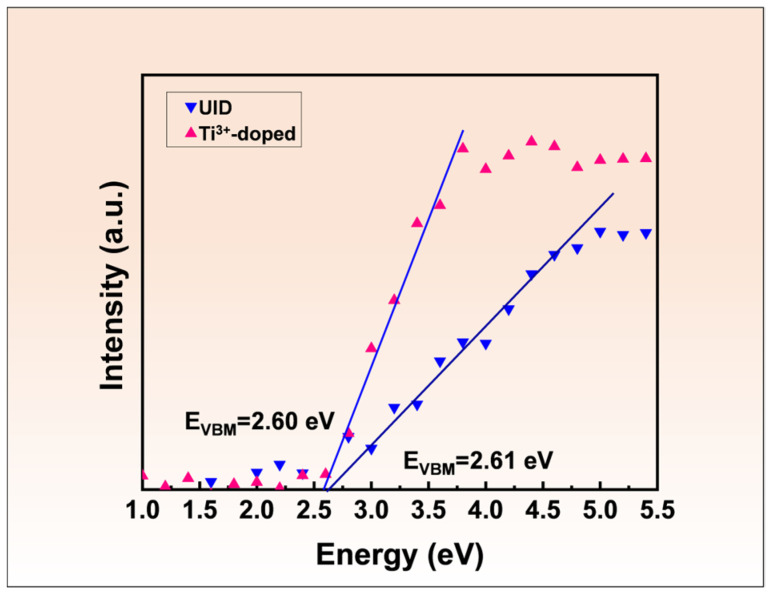
Comparison of the EVBM between Ti^3+^: *β*-Ga_2_O_3_ and Ti^3+^: *β*-Ga_2_O_3_.

The E_VBM_ measurement results for the UID and Ti^3+^: *β*-Ga_2_O_3_ crystals are shown in Figure 4, with values of 2.61 eV and 2.60 eV, respectively, which are very similar. Although the E_VBM_ of the Ti^3+^: *β*-Ga_2_O_3_ increased slightly, the magnitude of this shift is not comparable to the observed reduction in the bandgap. Furthermore, the composition and position of the E_VBM_ in *β*-Ga_2_O_3_ are primarily determined by the O 2p orbitals. During the doping, Ti^3+^ primarily substitutes for Ga^3+^, exerting minimal direct influence on the oxygen lattice. In conclusion, the main reason for the decreased bandgap in Ti^3+^: *β*-Ga_2_O_3_ is attributed to the downward shift of the conduction band minimum, caused by the intrinsically smaller bandgap of the dopant Ti_2_O_3_. The extent of the bandgap reduction is influenced by the doping concentration, which explains the relatively smaller decrease observed in our as-grown crystal.

**Figure 5:**
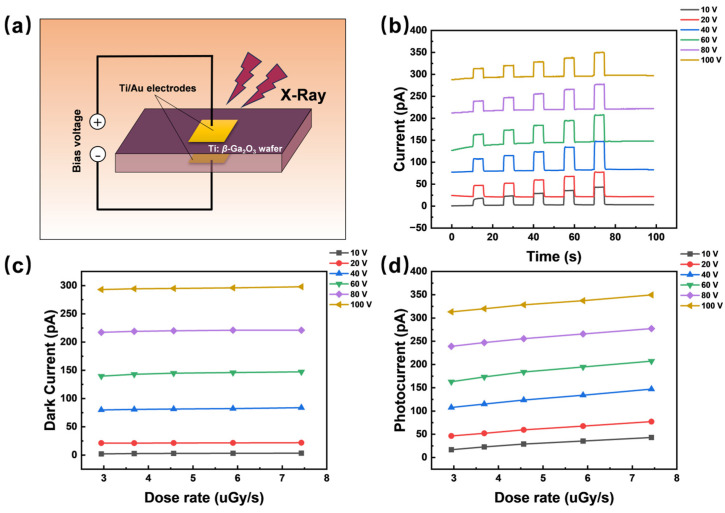
(**a**) Device schematic, (**b**) overall current response under different bias voltages and irradiation dose rates, (**c**) dark current trend, and (**d**) photocurrent trend.

Following the verification that Ti^3+^: *β*-Ga_2_O_3_ meets the performance requirements for X-ray detector applications, a (100)-oriented Ti^3+^: *β*-Ga_2_O_3_ wafer obtained via mechanical exfoliation was subjected to chemical mechanical polishing. Subsequently, Ti/Au electrodes with an area of 1 × 1 mm^2^ were deposited on both the front and back sides via evaporation. A rapid thermal annealing at 420 °C was then performed to lower the Schottky barrier and form ohmic contacts, enabling the collection of electrical signals generated upon X-ray irradiation. This process allowed for the fabrication of an MSM-structured X-ray detector. A schematic of the device is shown in Figure 5a.

To comprehensively evaluate the performance of the Ti^3+^: *β*-Ga_2_O_3_ X-ray detector, bias voltages ranging from 10 to 100 V were applied. The device response was measured under five different X ray dose rates, with the results shown in Figure 5b. It can be observed that, although the low doping concentration makes the dark current relatively sensitive to the increase in bias voltage, the photocurrent and dark current remain clearly distinguishable. This indicates that the concentration of deep level traps within the crystal, while low, is effective in suppressing the dark current without significantly affecting the photocurrent. The tube voltage of the X-ray generator used in this study was set to 40 kV, with tube currents of 10, 12.5, 16, 20, and 25 mA, corresponding to five distinct irradiation dose rates—2.944, 3.682, 4.567, 5.897, and 7.429 µGy/s—and were used to irradiate the device for equal durations. The corresponding dose rates at different time points are labeled in Figure 5b. The results show a clear positive correlation between the photocurrent and the irradiation dose rate under all bias conditions; that is, the photocurrent increases with a higher dose rate. This is because a higher dose rate delivers more energy, thereby generating more photocarriers. Consequently, the photocurrent was highest under the 7.429 µGy/s irradiation for all applied voltages. Figure 5c shows the trend of the dark current during X-ray exposure. While the dark current magnitude changes significantly with increasing bias voltage, it remains a flat line at a fixed voltage under different X ray dose rates. This demonstrates that the fabricated device can withstand irradiation at the tested intensities, indicating reliable operational stability. Figure 5d displays the current distribution under different X ray dose rates. Unlike the dark current, the photocurrent shows a distinct and increasing trend with rising dose rates. Overall, the current increases with applied voltage under all conditions, with no observed decrease at higher voltages. This indicates that the introduced defect concentration is at an appropriate level: it ensures device functionality while avoiding the generation of space charge under high bias and high dose rate conditions, which would otherwise degrade device performance.

**Figure 6:**
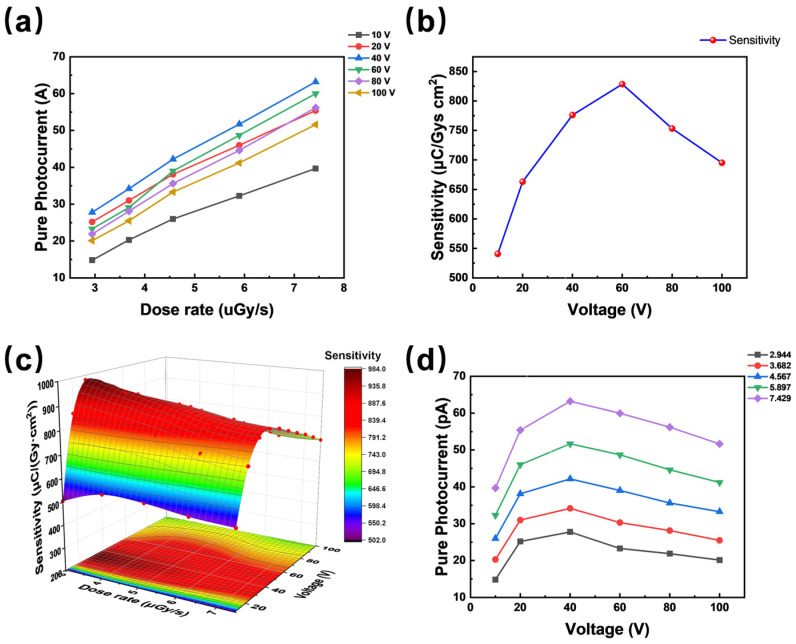
(**a**) Variation in the net photocurrent with irradiation dose under different bias voltages. (**b**) Device sensitivity at different bias voltages. (**c**) Sensitivity under various bias voltages and irradiation doses. (**d**) Variation in the net photocurrent with bias voltage under different irradiation doses.

As shown in Figure 6a, the irradiation dose rate and the net photocurrent exhibit an essentially linear relationship. The sensitivity at a fixed voltage can be calculated from the slope between them. The trend of sensitivity variation under different applied voltages is shown in Figure 6b, displaying an initial increase followed by a decrease. The sensitivity reached its maximum value of 828.50 μC/Gy·cm^2^ at 60 V, demonstrating the device’s excellent performance. It is noteworthy that the relationship between the net photocurrent and the irradiation dose in Figure 6a is not strictly linear but rather in a sublinear state. This is attributed to the deep-level defects introduced by Ti doping, which affect the carrier transport behavior. To analyze the sensitivity of the X-ray detector under specific operating conditions more comprehensively, it can be calculated using the following formula [32]:

S = (I_photo_ − I_dark_)/AD (3)

where S is the sensitivity, I_photo_ is the photocurrent, I_dark_ is the dark current, A is the effective irradiation area, and D is the irradiation dose rate. The sensitivity of the Ti^3+^: *β*-Ga_2_O_3_ X-ray detector under various bias voltages and irradiation dose rates is listed in Table 2. By creating a matrix of sensitivity, voltage, and irradiation dose rate, the surface plot in Figure 6c is generated, allowing for a clear visualization of the sensitivity trend. Unlike the overall current, which increases with both voltage and dose rate, the peak sensitivity does not occur at the highest voltage or dose rate. When the bias voltage is 40 V, the device sensitivity is generally higher than at other operating voltages, indicating that the net photocurrent behavior differs from the trend in the total current.

The relationship between the net photocurrent, bias voltage, and irradiation dose is shown in Figure 6d. The net photocurrent under each dose rate first increases and then decreases, reaching its maximum at 40 V. This trend corresponds to the observed sensitivity. It is noteworthy, however, that the sensitivity trend does not strictly follow the increase in net photocurrent. In Figure 6d, the net photocurrent increases consistently with higher irradiation doses, as a higher dose rate excites more photocarriers. In contrast, the sensitivity trend in Figure 6a shows that sensitivity is higher at relatively lower irradiation dose rates. Consequently, the device achieves its maximum sensitivity of 943.16 μC/(Gy·cm^2^) at 40 V and a dose rate of 2.944 µGy/s. This value represents the highest sensitivity reported for MSM-structured X-ray detectors fabricated from bulk *β*-Ga_2_O_3_ single crystals grown via semi-insulating or bandgap-engineering doping methods.

In summary, regarding overall detection capability, the Ti^3+^: *β*-Ga_2_O_3_-based X-ray detector exhibits optimal comprehensive performance at 60 V, with a sensitivity exceeding 800 μC/(Gy·cm^2^). Under the operating conditions of 40 V and 2.944 µGy/s, it achieves a peak sensitivity of 943.16 μC/(Gy·cm^2^), fully demonstrating the feasibility of fabricating high-performance X-ray detectors through the synergistic combination of bandgap engineering and defect-level modulation. Furthermore, the detector maintains a sensitivity exceeding 500 μC/(Gy·cm^2^) even at a bias as low as 10 V, indicating that it retains excellent detection capability at low operating voltages and is suitable for detection tasks under diverse working conditions.

**Figure 7:**
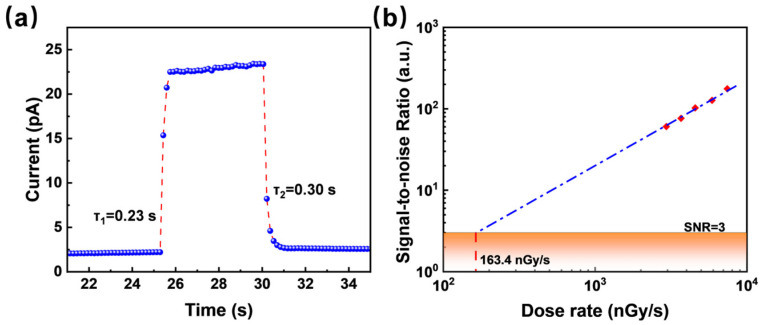
(**a**) Response time and (**b**) minimum detectable limit of the Ti^3+^: β-Ga_2_O_3_-based X-ray detector.

In addition to sensitivity, response time and minimum detectable limit are also critical metrics for evaluating detector performance. Figure 7a shows the response time of the Ti^3+^: *β*-Ga_2_O_3_-based detector, with a rise time (τ1) of 0.24 s and a fall time (τ2) of 0.30 s. This response is faster than that reported for detectors fabricated from UID crystals, which benefits from the lower concentration of V_O_. The presence of V_O_ affects carrier mobility; therefore, reducing its concentration effectively improves the response speed. However, compared to X-ray detectors made from *β*-Ga_2_O_3_ doped with other elements, the response time here is somewhat longer. This is attributed to the larger ionic radius of Ti^3+^ compared to Ga^3+^, leading to lattice expansion upon doping, which to some extent hinders carrier transport. Overall, however, the response time of the X-ray detector fabricated from Ti^3+^-doped *β*-Ga_2_O_3_ remains at a relatively excellent level.

The determination of the minimum detectable limit is related to the device’s signal-to-noise ratio (SNR). According to the standard established by the International Union of Pure and Applied Chemistry (IUPAC), a signal is considered reliable when SNR ≥ 3. Therefore, the irradiation intensity corresponding to SNR = 3 is defined as the MDL of the detector [33,34].

The SNR of an X-ray detector can be derived from the formula [35]:

SNR = I_photo_ − I_dark_/(1/N∑^n^_i_(I_i_ − I_photo_)^2^)^1/2^ (4)

where SNR is the signal-to-noise ratio, I_photo_ is the photocurrent, and I_dark_ is the dark current. The numerator represents the net photocurrent, while the entire denominator represents the standard deviation of the photocurrent. Based on the sensitivity calculations, the device exhibits optimal performance for low-dose-rate X-ray detection at 40 V. Therefore, the current data obtained at this bias voltage was used for the SNR calculation. As the irradiation intensity increases, the net photocurrent necessarily increases, yielding a roughly linear relationship between the overall SNR and irradiation intensity, as shown in Figure 7b. By performing a linear fit on the calculated SNR values, the irradiation dose rate corresponding to SNR = 3 can be extrapolated. The calculated minimum detectable limit for our fabricated X-ray detector is 164.26 nGy/s, demonstrating its capability to detect low-dose-rate radiation.

Due to the moderate doping concentration, the crystal’s resistivity increased, effectively suppressing the dark current, while the band gap narrowed slightly. This strategy yielded a dual benefit: it alleviated the suppression of photocurrent by trap states and simultaneously circumvented the typical increase in dark current associated with bandgap reduction, thereby fully leveraging the gain in photogenerated carrier density. Overall, the enhancement in the crystal’s detection performance was primarily attributable to the modification of its electrical properties induced by Ti^3+^ incorporation. Facilitated by bandgap engineering, more favorable outcomes were achieved, enabling the device to function efficiently even under a low applied bias. As a result, Ti^3+^-doped *β*-Ga_2_O_3_ offers several advantages for X-ray detection applications: ease of device fabrication, low operating voltage, high sensitivity, fast response, and a low detection limit.

## 4. Conclusions

Overall, this study successfully grew high-quality Ti^3+^: *β*-Ga_2_O_3_ single crystals using the EFG method and compared their optoelectronic properties with those of the UID crystal. It was confirmed that the incorporation of Ti^3+^ effectively increased the resistivity of the crystal and reduced its bandgap to some extent. The alloying of Ga_2_O_3_ with Ti_2_O_3_ and the lattice expansion induced by Ti^3+^ were responsible for the narrowing of the bandgap. Doping with Ti^3+^ led to a slight decrease in oxygen vacancy concentration within the crystal but had little effect on the value of the E_VBM_. An MSM structured X-ray detector based on Ti^3+^: *β*-Ga_2_O_3_ was fabricated, demonstrating a sensitivity of 945.16 μC/(Gy·cm^2^) under a bias voltage of 40 V and an exposure dose rate of 2.944 μGy/s. Moreover, even at a low bias of 10 V, the sensitivity still exceeded 500 μC/(Gy·cm^2^). The detector exhibited response times of 0.24 s (τ1) and 0.30 s (τ2), with an MDL of 164.26 nGy/s. These combined results demonstrate that, under the dual regulation of band engineering and electrical modulation, Ti^3+^: *β*-Ga_2_O_3_ enables highly sensitive X-ray detection at low voltages and low dose rates, highlighting its strong application potential.

## Figures and Tables

**Table 1:** Comparison of bandgap widths for Ga_2_O_3_ with different doping elements and crystal planes.

Crystal	Plane	Bandgap	Ref.
UID *β*-Ga_2_O_3_	(100)	4.70	[25]
UID *β*-Ga_2_O_3_	(010)	4.55	[25]
UID *β*-Ga_2_O_3_	(001)	4.70	[25]
UID *β*-Ga_2_O_3_	(100)	4.76	This work
Fe: *β*-Ga_2_O_3_	(100)	4.72	[9]
Mg: *β*-Ga_2_O_3_	(100)	4.72	[10]
Ni: *β*-Ga_2_O_3_	(100)	4.74	[11]
Ti: *β*-Ga_2_O_3_	(100)	4.59	This work

**Table 2:** Sensitivity under different biases and irradiation dose rates.

Voltage (V)	Dose Rate (μGy/s)	Sensitivity (μC/(Gy·cm^2^))	Voltage (V)	Dose Rate (μGy/s)	Sensitivity (μC/(Gy·cm^2^))
10	2.944	504.44	20	2.944	855.45
	3.682	551.12		3.682	841.65
	4.567	568.91		4.567	834.57
	5.897	547.64		5.897	780.24
	7.429	533.97		7.429	745.37
40	2.944	943.16	60	2.944	790.38
	3.682	927.85		3.682	822.92
	4.567	923.02		4.567	854.43
	5.897	876.32		5.897	825.67
	7.429	851.03		7.429	807.26
80	2.944	742.52	100	2.944	683.73
	3.682	876.32		3.682	851.03
	4.567	779.79		4.567	728.27
	5.897	756.54		5.897	698.55
	7.429	756.12		7.429	695.18

## Data Availability

The original contributions presented in this study are included in the article. Further inquiries can be directed to the corresponding author.

## Abbreviations

The following abbreviations are used in this manuscript:

Ga_2_O_3_	Gallium oxide
EFG	Edge-defined Film-fed Growth
HRXRD	High-resolution X-ray diffraction
FWHM	Full width at half maximum
UID	Unintentionally Doped
XPS	X-ray Photoelectron Spectroscopy
E_VBM_	Valence Band Maximum
MSM	Metal–Semiconductor–Metal
MDL	Minimum Detectable Limit
CT	Computed Tomography
UV–vis	Ultraviolet–visible
IR	Infrared
SNR	Signal-to-Noise Ratio
